# Preditores de Mortalidade Intra-Hospitalar de Pacientes com Infarto Agudo do Miocárdio com Choque Cardiogênico em Uso de Balão Intra-Aórtico

**DOI:** 10.36660/abc.20230496

**Published:** 2025-02-04

**Authors:** Rossana Dall’Orto Elias, Isabella Pedrosa Assunção, Julliane Vasconcelos Joviano Santos, Maria da Gloria Rodrigues-Machado, José Luiz Barros Pena

**Affiliations:** 1 Biocor Instituto Nova Lima Brasil Biocor Instituto, Nova Lima, MG – Brasil; 2 Faculdade de Ciências Médicas de Minas Gerais Belo Horizonte MG Brasil Faculdade de Ciências Médicas de Minas Gerais, Belo Horizonte, MG – Brasil; 3 Hospital Felicio Rocho Belo Horizonte MG Brasil Hospital Felicio Rocho - Ecocardiografia, Belo Horizonte, MG – Brasil

**Keywords:** Choque Cardiogênico, Balão Intra-aórtico, Infarto do Miocárdio com Supradesnível do Segmento ST, Mortalidade Hospitalar

## Abstract

**Fundamento:**

Pacientes com infarto agudo do miocárdio com supradesnivelamento do segmento ST (IAMCSST) e choque cardiogênico (CC) têm elevado risco de morte. Novos tipos de dispositivos mecânicos têm limitada disponibilidade em nosso meio. O uso de balão intra-aórtico (BIA), apesar da indicação rebaixada em novas diretrizes, constitui a estratégia de suporte mecânico mais empregada. Entretanto, os preditores clínicos de sua efetividade na redução de morte nesse grupo são pouco conhecidos.

**Objetivos:**

Avaliar os preditores de efetividade do BIA na redução da mortalidade intra-hospitalar de pacientes com IAMCSST e CC.

**Métodos:**

Estudo observacional, retrospectivo, descritivo, unicêntrico, envolvendo 98 pacientes com IAMCSST e CC que utilizaram BIA, na unidade de terapia intensiva. Comparamos os pacientes que sobreviveram (42 homens e 13 mulheres) ou não (30 homens e 13 mulheres) através dos preditores clínicos de efetividade do BIA na redução de morte intra-hospitalar, considerando um nível de significância estatística de 5% (p < 0,05).

**Resultados:**

O emprego de BIA em pacientes com menos de um dia de infarto (odds ratio [OR]: 0,12; intervalo de confiança [IC] de 95%: 0,02 a 0,85; p = 0,034) constituiu fator de aumento do risco de morte intra-hospitalar. Pacientes mais jovens (OR: 1,09; IC 95%: 1,02 a 1,16; p = 0,010) e dislipidêmicos (OR: 0,19; IC 95%: 0,05 a 0,81; p = 0,024) constituíram preditores de redução de morte intra-hospitalar. A cada ano a mais na idade, o risco de óbito aumentou 1,07 vezes.

**Conclusão:**

Em pacientes com IAMCSST e CC, o uso de BIA reduziu mortalidade intra-hospitalar quando foi utilizado por 2 ou mais dias e em pacientes mais jovens e dislipidêmicos. Estudos adicionais são necessários para confirmar esses achados.

## Graphical abstract

**Figura Central:**
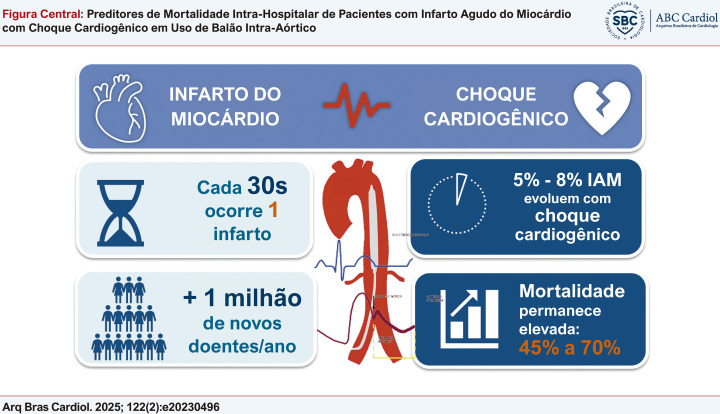
Preditores de Mortalidade Intra-Hospitalar de Pacientes com Infarto Agudo do Miocárdio com Choque Cardiogênico em Uso de Balão Intra-Aórtico

**Figura Central:** Preditores de Mortalidade Intra-Hospitalar de Pacientes com Infarto Agudo do Miocárdio com Choque Cardiogênico em Uso de Balão Intra-Aórtico

*IAM: infarto agudo do miocárdio.*

## Introdução

O infarto agudo do miocárdio (IAM) é a causa mais frequente de choque cardiogênico,^1^ com incidência entre 5% e 15%^2^ e elevada mortalidade, superior a 50%.^3^

**Figura 1:**
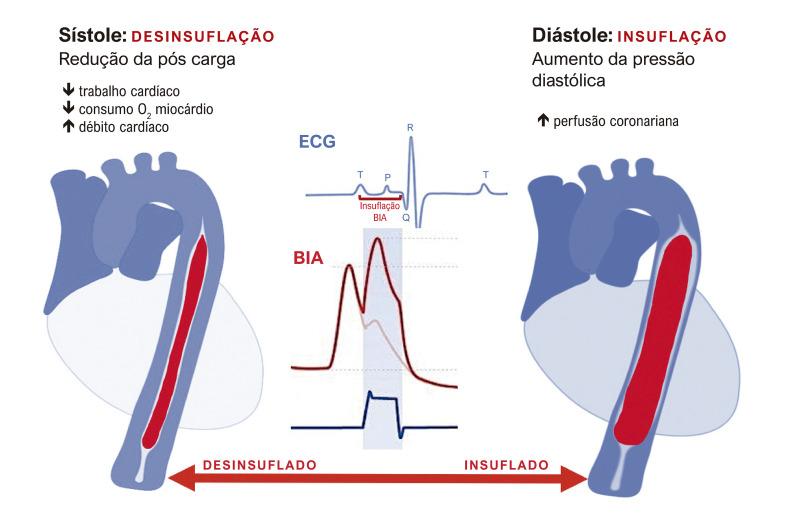
– Representação esquemática do BIA em sístole e diástole com traçado de ECG e traçados correspondentes do BIA. O dispositivo funciona para inflar durante a diástole (à direita) e desinsuflar durante a sístole. Isto pode ser conseguido cronometrando o tempo em relação ao ECG ou às ondas de pressão, ilustradas acima, para inflar com precisão durante a porção apropriada do ciclo cardíaco. A forma de onda do BIA, ilustrada em azul, é cronometrada para se correlacionar verticalmente com a diástole nos traçados arteriais e do ECG. BIA: balão intra-aórtico; ECG, eletrocardiograma.

O balão intra-aórtico (BIA) permanece como suporte ainda muito utilizado em vários serviços de cardiologia, embora a sua substituição venha ocorrendo com maior frequência.^4,5^ Este dispositivo auxilia o coração diminuindo indiretamente a pós-carga e aumentando a pressão diastólica na raiz da aorta. Estes efeitos aumentam o fluxo sanguíneo coronariano, resultando em melhor perfusão. Os efeitos cardiovasculares do BIA são devidos às ações na pré e pós-carga, com diminuição da pressão arterial sistólica em até 10% e da pressão aórtica diastólica final em até 30%. Há também aumento na fração de ejeção do ventrículo esquerdo (FEVE) com aumento do débito cardíaco entre 0,5 e 1 L/min ou até 30%.^6-8^ O mecanismo de ação do BIA deriva do conceito de contra pulsação: insuflação diastólica e rápida deflação sistólica. O aumento do volume na aorta durante a diástole resulta na melhoria da circulação coronariana com redistribuição do fluxo sanguíneo aumentando a perfusão coronariana. A rápida deflação leva a uma redução da pós-carga (Figura 1). Esses mecanismos teoricamente proporcionam um aumento no suprimento do oxigênio ao mesmo tempo em que reduzem o consumo de oxigênio pelo miocárdio.^9,10^

O choque cardiogênico pós-IAM tem sido a principal indicação de BIA por anos. Entretanto, os resultados do estudo IABP-SHOCK II em 2012, o maior estudo relacionado ao BIA, causou um declínio significativo em seu uso.^5,11^ Este estudo mostrou que não houve diferença entre os dois grupos na mortalidade por todas as causas após 30 dias do IAM e tampouco com relação às taxas de reinfarto, repetição da revascularização, acidente vascular cerebral, sepse, complicações isquêmicas periféricas, insuficiência renal e sangramento importante. Apesar dos efeitos neutros do BIA no choque cardiogênico do paciente com infarto agudo do miocárdio com supradesnivelamento do segmento ST (IAMCSST), a análise de subgrupo do ensaio IABP-SHOCK II revelou que pacientes jovens sem IAM prévio, não hipertensos se beneficiaram do BIA.

Apesar do surgimento de outros dispositivos de suporte circulatório mecânico, como oxigenação por membrana extracorpórea venoarterial (VA-ECMO), o BIA apresenta vantagens técnicas como facilidade de implantação, maior familiaridade da equipe médica, menor custo e menores complicações em comparação com outros modelos.^10^

Mesmo com as alterações nas diretrizes de uso do BIA, estudos ainda são necessários, já que esse dispositivo teoricamente proporciona um aumento na relação de oferta/demanda de oxigênio, resultando em maior viabilidade endocárdica.^6^ O objetivo deste estudo é avaliar preditores de mortalidade intra-hospitalar de pacientes que utilizaram BIA no IAMCSST e identificar subgrupos que beneficiariam de sua utilização.

## Materiais e métodos

Trata-se de um estudo observacional, transversal, retrospectivo, descritivo e analítico, em centro único. Foram avaliados 98 pacientes internados no Biocor Instituto com diagnóstico de IAMCSST no período de janeiro de 2005 a abril de 2022. Foram incluídos pacientes que evoluíram com choque cardiogênico após IAMCSST e utilizaram o BIA. Os critérios de exclusão foram dilatação aneurismática da aorta; pós-operatório de cirurgia de aorta ascendente e descendente; presença de insuficiência aórtica de grau moderado a importante; pacientes com pós-parada cardíaca que obtiveram retorno à circulação espontânea, mas tiveram desfecho neurológico desfavorável; infarto isolado do ventrículo direito; doença arterial periférica grave; e pacientes com enxerto em artérias femorais (*bypass*).

O IAM foi definido como dor torácica persistente com detecção de aumento ou queda dos níveis de marcadores de injúria miocárdica (com pelo menos um valor acima do percentil 99). Um dos 5 critérios deveria estar presente para que o diagnóstico de infarto fosse confirmado: (1) sintomas de isquemia miocárdica; (2) alterações do segmento ST/onda T ou bloqueio completo de ramo esquerdo novos; (3) desenvolvimento de ondas Q patológicas no eletrocardiograma; (4) perda de músculo miocárdico viável ou alteração de motilidade segmentar por exame de imagem; (5) identificação de trombo intracoronário por angiografia ou autópsia.^12,13^

O choque cardiogênico foi definido clinicamente por hipotensão (pressão arterial sistólica < 90 mmHg por > 30 min ou necessidade de administração contínua de vasopressores por > 30 min a fim de manter a pressão arterial sistólica > 90 mmHg, apesar da carga volêmica adequada, além de hipoperfusão em órgão-alvo [extremidades frias ou débito urinário < 30 mL/h]), sinais radiológicos de congestão pulmonar e elevação da concentração sérica de lactato.^14,15^

As características compostas por sexo, idade, incluindo histórico médico de comorbidades como hipertensão arterial sistêmica (HAS), diabetes mellitus (DM), tabagismo, dislipidemia, IAM prévio, história familiar para doença coronariana,^16^ escores de risco como classificação de Killip-Kimball^17^ e TIMI Risk,^18^ avaliação da função ventricular esquerda pela ecocardiografia, avaliação da artéria coronária culpada pelo IAM, tempo porta-balão, tempo de utilização do suporte circulatório (BIA) e óbito, foram incluídas na coleta dos registros médicos.

HAS foi definida como pressão sistólica > 140 mmHg ou pressão diastólica > 90 mmHg durante o exame físico, ou pelo uso de medicamentos anti-hipertensivos. DM foi definido como glicose de jejum > 126 mg/dL, ou uso de insulina ou hipoglicemiantes orais. Tabagistas foram definidos como fumantes ativos no momento da admissão hospitalar ou que pararam de fumar nos últimos 6 meses. Dislipidemia foi definida como colesterol sérico total > 200 mg/dL ou pelo uso de estatina. História de doença aterosclerótica coronariana foi definida como IAM prévio à admissão ou qualquer intervenção vascular anterior.^19^

A avaliação da função ventricular esquerda foi feita por meio do cálculo da FEVE pelo método de Simpson e foram coletados os resultados provenientes do primeiro ecocardiograma transtorácico realizado nos pacientes com IAMCSST após admissão no hospital. A disfunção ventricular esquerda foi definida como FEVE menor ou igual a 40%.

A coleta de dados foi realizada após aprovação do projeto pelo Comitê de Ética Médica e Comitê de Ética em Pesquisa (CEP) da Faculdade Ciências Médicas, dentro dos preceitos éticos, na observância total às regras da pesquisa para a realização de estudos, com respeito ao sigilo profissional e à não exposição da identidade do paciente, sem ocasionar danos físicos ou morais à integridade destes (CAAE: 49871221.4.0000.5134).

### Análise estatística

Os dados foram apresentados em tabelas contendo as frequências absolutas e suas respectivas porcentagens assim como média ± desvio-padrão ou mediana e intervalo interquartil para variáveis contínuas com e sem distribuição normal, respectivamente. As variáveis contínuas foram testadas quanto à normalidade pelo Teste de Kolmogorov-Smirnov. Para a análise bivariada, considerando o óbito como desfecho, foram utilizados os testes t de Student não pareado e Mann-Whitney para as variáveis contínuas de idade e tempo porta-balão, respectivamente. Para as variáveis categóricas foram utilizados os testes de qui-quadrado, o teste exato de Fisher. A simulação de Monte Carlo foi utilizada para mais de 2 categorias de resposta em frequências inferiores a 5. Em todos os testes, o nível de significância adotado foi 5%, portanto, consideradas significativas as comparações cujo valor p estivesse inferior a 5%.

Para determinar os fatores que em conjunto estavam associados ao óbito, foi realizado um modelo multivariado de regressão logística (*backward stepwise*). Nesta etapa, foram selecionadas para inclusão no modelo logístico multivariado inicial todas as variáveis que apresentaram valor p < 0,20 na análise bivariada. Permaneceram no modelo logístico multivariado final as variáveis que apresentaram nível de significância estatística (p < 0,05) e *odds ratio* (OR) significativa de acordo com intervalo de confiança (IC) de 95%. Variáveis que apresentavam mais que duas categorias foram transformadas em variáveis “*dummies*”. As variáveis que apresentaram colinearidade foram avaliadas e retiradas do modelo. Para definição do modelo final, foi utilizado o teste da razão da verossimilhança. O desempenho do modelo foi avaliado pelo teste de Hosmer-Lemeshow.

As análises do presente estudo foram realizadas utilizando o SPSS, versão 25.0 juntamente com recursos do Microsoft Excel (editor de planilhas).

## Resultados

Entre o período de janeiro de 2005 e abril de 2022 foram selecionados 98 prontuários de pacientes que evoluíram com choque cardiogênico após IAMCSST e fizeram uso de BIA, em uma única instituição do Brasil.

Na Tabela 1 são apresentadas as características da amostra estudada, sendo a maioria do sexo masculino (73,5%) com média de idade de 66,5 ± 12,3 anos (variação de 37 a 93 anos). O tempo porta-balão médio foi de 60 ± 25,6 minutos, variando de 20 a 180 minutos. A comorbidade mais frequente dos pacientes foi a HAS, que esteve presente em 70 pacientes (73,7%). Tabagismo e antecedentes familiares corresponderam, respectivamente, a 37,8% e 31,2%.

**Tabela 1:** – Características dos pacientes do estudo

Variáveis	n (%)
**Sexo**	
Masculino	72/98 (73,5%)
Feminino	26/98 (26,5%)
**Idade (em anos)**	
Média (DP)	66,5 (12,3)
Mediana	65,5
**Tempo porta-balão (em minutos)**	
Média (DP)	60 (25,6)
Mediana	60
**Comorbidades**	
HAS	70/95 (73,7%)
Dislipidemia	38/81 (46,9%)
Diabetes	34/95 (35,8%)
IAM prévio	12/95 (12,6%)
Tabagismo	34/95 (37,8%)
**Antecedentes familiares de insuficiência coronariana**	24/77 (31,2%)

*Os dados estão apresentados em média (DP) e mediana. DP: desvio padrão; HAS: hipertensão arterial sistêmica; IAM: infarto agudo do miocárdio.*

A maioria dos pacientes com IAMCSST hipotensos à admissão submetidos ao implante do BIA estavam em Killip IV (39,2%). A artéria culpada mais frequente foi a descendente anterior em 80% dos pacientes e 95 pacientes (95,9%) evoluíram com disfunção ventricular. O BIA foi implantado no mesmo dia do IAM em 73 pacientes (74,5%) e a maioria fez uso do dispositivo por 3 ou mais dias (46,9%) (Tabela 1).

A cirurgia de revascularização miocárdica (CRVM) de urgência como forma de revascularização foi utilizada em 4% dos pacientes (Tabela 1), demonstrando sucesso em todos os casos (Tabela 2). A porcentagem total de óbitos atingiu 43,9% e de alta hospitalar 55,7% (Tabela 1).

**Tabela 2:** – Características e evolução dos pacientes

Variáveis	n (%)
**Killip**	
I	18/97 (18,6%)
II	27/97 (27,8%)
III	14/97 (14,4%)
IV	38/97 (39,2%)
**CRVM de urgência**	4/98 (4,1%)
**Disfunção ventricular**	57/87 (66%)
**Alta hospitalar**	54/97 (55,7%)
**Artéria culpada**	
Descendente anterior	72/90 (80%)
Circunflexa	7/90 (7,8%)
Coronária direita	11/90 (12,2%)
**Dias do IAM até o implante BIA**	
0 dia	73/98 (74,5%)
1 dia	14/98 (14,3%)
2 dias	4/98 (4,1%)
3 ou mais	7/98 (7,1%)
**Dias de uso do BIA**	
0 a 1 dia	13/98 (13,3%)
2 a 3 dias	39/98 (39,8%)
3 ou mais	46/98(46,9%)
**Óbito**	43/98 (43,9%)

*Os dados estão apresentados em números absolutos e (porcentagem). BIA: balão intra-aórtico; CRVM: cirurgia de revascularização do miocárdio; IAM: infarto agudo do miocárdio.*

**Figura 2:**
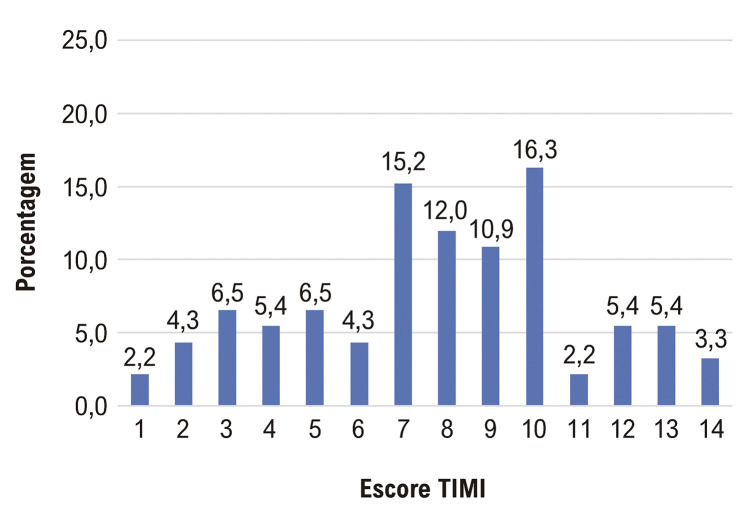
– Classificação do escore de risco TIMI.

A Figura 2 representa a classificação do escore de risco TIMI dos pacientes avaliados, demonstrando que 50% apresentaram resultado maior que 8 (mediana = 8; intervalo interquartil = 5 a 10), representando um risco de morte em 30 dias maior que 26,8% de acordo com os índices do escore.

Analisando o desfecho primário composto por óbito intra-hospitalar e correlacionando-o com as variáveis estudadas, foi evidenciado que para sexo, tempo porta-balão, IAM prévio, DM, HAS, tabagismo, dislipidemia, antecedentes familiares de insuficiência coronariana, CRVM de urgência, disfunção ventricular, artéria culpada e dias de IAM até o implante do BIA, não foi constatada diferença estatisticamente significativa (p ≥ 0,05), ou seja, não houve associação destas variáveis analisadas com o desfecho óbito (Tabela 3).

**Tabela 3:** – Avaliação das variáveis do estudo de acordo com o desfecho (óbito)

Variáveis	Óbito		Valor p
	Não (n=55)	Sim (n=43)	
**Sexo**			
Masculino	42 (76,4%)	30 (69,8%)	0,496^q^
Feminino	13 (23,6%)	13 (30,2%)	
Idade (anos)*			
< 50 anos	7	0	0,017^q^
50 a 75 anos	35	29	
> 75 anos	3	14	
Tempo porta-balão (min)**	50 (45 - 70)	60 (42 - 80)	0,438
**IAM prévio**			
Não	45 (84,9%)	38 (90,5%)	0,540^q^
Sim	8 (15,1%)	4 (9,5%)	
**Diabetes**			
Não	34 (64,2%)	27 (64,3%)	> 0,999^q^
Sim	19 (35,8%)	15 (35,7%)	
**Hipertensão arterial sistêmica**			
Não	12 (22,6%)	13 (31%)	0,482^q^
Sim	41 (77,4%)	29 (69%)	
**Tabagismo**			
Não	31 (62%)	25 (62,5%)	> 0,999^q^
Sim	19 (38%)	15 (37,5%)	
**Dislipidemia**			
Não	19 (44,2%)	24 (63,2%)	0,119^q^
Sim	24 (55,8%)	14 (36,8%)	
**Antecedentes familiares de insuficiência coronariana**			
Não	24 (60%)	29 (78,4%)	0,092^q^
Sim	16 (40%)	8 (21,6%)	
**CRVM de urgência**			
Não	51 (92,7%)	43 (100%)	0,129^f^
Sim	4 (7,3%)	0 (90,0%)	
**Killip**			
I, II e III	38 (70,4%)	21 (48,8%)	**0,031^**q**^**
IV	16 (29,6%)	22 (51,2%)	
**Disfunção ventricular**			
Não	25 (35%)	5 (42%)	0,115^f^
Sim	47 (65%)	7 (58%)	
**Artéria culpada**			
Descendente anterior	43 (86%)	29 (72,5%)	0,238^mc^
Circunflexa	2 (4%)	5 (12,5%)	
Coronária direita	5 (10%)	6 (15%)	
**Dias do infarto até implante BIA**			
0 dias	37 (67,3%)	36 (83,7%)	0,053^mc^
1 dia	12 (21,8%)	2 (4,7%)	
2 ou mais dias	6 (10,9%)	5 (11,6%)	
**Dias de uso do BIA**			
0 a 1	1 (1,8%)	12 (27,9%)	**0,001^**mc**^**
2 a 3 dias	25 (45,5%)	14 (32,6%)	
Mais que 3	29 (52,7%)	17 (39,5%)	

BIA: balão intra-aórtico; CRVM: cirurgia de revascularização do miocárdio; f: teste exato de Fisher; IAM: infarto agudo do miocárdio; mc: qui-quadrado com simulação de Monte Carlo; q: qui-quadrado de Pearson. * Dados representados em frequência; ** Dados representados em mediana (P25 a P75).

Por outro lado, em relação aos dias de uso do BIA, os grupos óbito e não óbito apresentaram diferença estatisticamente significativa (p < 0,001), sendo evidenciado menor percentual de pacientes com o desfecho óbito a partir de 2 ou mais dias de uso em relação aos pacientes que utilizaram pelo período de 0 a 1 dia (Tabela 3).

A 4 apresenta o modelo de regressão logística multivariado para o desfecho primário composto por óbito intra-hospitalar, indicando quais os fatores em conjunto foram associados ao desfecho. O modelo inicial apresentou todas as variáveis com valor p < 0,20, exceto idade, dislipidemia e dias do IAM até implante do BIA.

A cada ano a mais na idade, houve aumento de 1,07 vezes na chance de óbito. No entanto, pacientes com dislipidemia e pacientes com implante de BIA 1 dia após o infarto apresentaram redução no risco de óbito, sendo este comparado aos pacientes que usaram BIA no momento do diagnóstico (dia 0) (Tabela 4).

**Tabela 4:** – Modelo de regressão multivariado para o desfecho óbito

		Modelo cheio			
	Coeficiente B	Valor p	OR	IC 95% para OR	
Variáveis					
Idade	0,08	0,010	1,09	1,02	1,16
Dislipidemia	−1,65	0,024	0,19	0,05	0,81
Antecedentes familiares de insuficiência coronariana	−0,47	0,529	0,63	0,15	2,69
CRVM de urgência	−21,23	0,999	0,00	0,00	
Killip	0,339	0,510	1,40	0,50	3,93
Dias de infarto (0 – categoria referência)		0,052			
Dias de infarto (1 dia)	−2,16	0,034	0,12	0,02	0,85
Dias de infarto (2 ou mais dias)	0,85	0,456	2,34	0,25	21,87
Dias de uso do BIA (0 ou 1 dia – categoria referência)		0,921			
Dias de uso do BIA (2 a 3 dias)	−20,91	0,999	0,00	0,00	
Dias de uso do BIA (mais de 3 dias)	−21,18	0,999	0,00	0,00	
Constante	17,11	0,999	2699,61		
		**Modelo final**			
	**Coeficiente B**	**Valor p**	**OR**	**IC 95% para OR**	
Idade	0,07	0,005	1,07	1,02	1,13
Dislipidemia	−1,58	0,005	0,21	0,07	0,63
Dias de infarto (0, categoria referência)		0,008			
Dias de infarto (1 dia)	−2,91	0,002	0,05	0,01	0,34
Dias de infarto (2 ou mais dias)	−0,44	0,579	0,64	0,13	3,07
Constante	−3,69	0,020	0,03		

*Teste de Hosmer-Lemeshow: p = 0,976; pseudo-R = 0,317; percentual de classificação correta = 72,8%. BIA: balão intra-aórtico; CRVM: cirurgia de revascularização do miocárdio; IC: intervalo de confiança; OR: odds ratio.*

## Discussão

O estudo apresentou como objetivo principal identificar as características clínicas associadas ao prognóstico do uso de BIA em pacientes com IAMCSST que evoluíram para choque cardiogênico. Diferentemente do estudo IABP-SHOCK II,^1^ que avaliou a mortalidade em 30 dias e durante o seguimento de 6,2 anos, este estudo limitou-se ao período intra-hospitalar, sem menção ao impacto na sobrevida após a alta hospitalar.

Como foi demonstrado, preditores como sexo, tempo porta-balão, IAM prévio, dias de IAM até o implante do BIA, DM, HAS, tabagismo, dislipidemia, antecedentes familiares de insuficiência coronariana, CRVM de urgência, disfunção ventricular e artéria coronária culpada não foram determinantes no impacto da mortalidade intra-hospitalar.

Similarmente ao estudo CULPRIT-SHOCK,^20^ no presente estudo a artéria descendente anterior foi a mais prevalente nos casos de choque cardiogênico, provavelmente porque se relaciona com grande quantidade de músculo miocárdio comprometido quando ocluída. Apesar desse músculo em risco, classificado pela FEVE, a qual denominamos no estudo como disfunção ventricular, ser identificado em quase todos os pacientes do grupo choque cardiogênico, essa variável não apresentou fator de impacto para óbito.

O escores de risco TIMI^17^ e Killip-Kimball^17,18^ foram calculados na admissão e o implante do BIA foi realizado após a evolução para choque cardiogênico. Nossos dados mostraram que dos pacientes admitidos em choque cardiogênico na vigência do IAMCSST e implante de BIA, 51,4% evoluíram para óbito, enquanto, comparativamente, no escore Killip-Kimball, a mortalidade foi 81% com implante de BIA. Assim como o estudo IABP-SHOCK II,^1^ este trabalho não especifica a gravidade do quadro clínico e nem classifica o choque cardiogênico dos pacientes em que foi implantado o BIA, podendo este fato estar relacionado à alta mortalidade no implante do dispositivo ao diagnóstico e à alta mortalidade de pacientes que usaram BIA por menos de 2 dias, conforme demonstrado a partir da análise estatística nas Tabelas 3 e 4.

Quanto ao tempo de ocorrência do IAMCSST e o implante do BIA, foi observado que em 73 pacientes (74,5% da amostra) o implante ocorreu nas primeiras 24 horas e, destes, 36 faleceram. Estes dados confirmam os achados da literatura quanto à mortalidade elevada.^21^

Comparativamente com ensaios clínicos controlados aleatorizados e análises com dispositivos de contrapulsação, como citados pelos trabalhos de Vallabhajosyula et al.^11^ e Koenig et al.,^7^ os estudos não mostraram superioridade de outros dispositivos em relação ao BIA, este podendo ser a opção de escolha, principalmente em países em desenvolvimento.

Na nossa amostra, encontramos 43 pacientes que utilizaram BIA por mais de 3 dias. A mortalidade foi maior nesse grupo do que nos que utilizaram por até 3 dias. Este resultado está provavelmente relacionado à gravidade da evolução e manutenção do choque cardiogênico, persistindo a disfunção ventricular com necessidade do uso de vasopressores.^22^

O BIA foi introduzido na prática clínica há 5 décadas e continua sendo um dos dispositivos de suporte mais comum usado em choque cardiogênico em nosso meio.^23^ Acredita-se que o BIA diminua o consumo de oxigênio pelo miocárdio, aumente a perfusão das artérias coronárias, diminua a pós-carga e aumente modestamente o débito cardíaco (0,8 a 1 L/min).^22^ Existem vários dispositivos de assistência ventricular, no entanto, os mais comumente usados no choque cardiogênico são os dispositivos Impella e BIA. O Impella atua independentemente da função e do ritmo cardíaco e, à medida que a taxa de fluxo cardíaco aumenta, ele alivia progressivamente o ventrículo esquerdo e, consequentemente, o consumo de oxigênio pelo miocárdio.^22^

O estudo IMPRESS in Severe Shock comparou aleatoriamente o uso de Impella *versus* BIA em pacientes com IAM associado ao choque cardiogênico. O desfecho primário foi a mortalidade em 30 dias e o estudo não encontrou diferença significativa na mortalidade em 30 dias (cerca de 50% para ambos os grupos).^24^

Os estudos SHOCK,^24^ IABP-SHOCK II^1^ e o IMPRESS em choque cardiogênico grave^24^ mostraram aproximadamente 50% de mortalidade em 6 a 12 meses, elucidando os resultados constantes de mortalidade em choque cardiogênico nas últimas 2 décadas, apesar do uso generalizado de dispositivos de suporte circulatório mecânico. Uma análise recente do registro cVAD (dispositivo de assistência ventricular baseado em cateter) indica que o implante precoce de suporte circulatório mecânico em pacientes com choque cardiogênico, antes de iniciar o suporte inotrópico/vasopressor e antes da angioplastia, está independentemente associado a melhores taxas de sobrevida em pacientes com choque devido a IAM.^25^

Na literatura ainda faltam dados sobre o perfil clínico e hemodinâmico dos pacientes que utilizaram e se beneficiariam do uso de BIA, além de um seguimento pós alta hospitalar, visando não somente avaliar a mortalidade intra, mas também peri e pós-hospitalar do choque cardiogênico.

Há ainda importantes distinções a serem analisadas futuramente para avaliar a eficácia dos dispositivos de suporte circulatórios, como a gravidade do choque cardiogênico, tendo como modelo sugerido uma classificação em 5 estágios pela Sociedade de Intervenção e Angiografia Cardiovascular nos Estados Unidos,^26,27^como forma de estratificar o risco e definir qual paciente se beneficiaria do uso dos dispositivos de contrapulsação. Estudos para avaliação e seguimento do uso do BIA têm sido publicados na literatura médica com mais frequência,^5^ alguns destes são divergentes do grande estudo IABP-SHOCK II, que promoveu o rebaixamento na indicação do dispositivo nas últimas diretrizes.^13^ Entretanto, esses novos estudos ainda se mostram escassos em avaliar a precocidade do implante do dispositivo e de uma definição clínica e universal da classificação do choque cardiogênico para avaliar os fatores para melhora do prognóstico e redução da mortalidade intra-hospitalar e a longo prazo. Embora outros dispositivos de suporte circulatório mecânico tenham sido desenvolvidos, o BIA continua muito utilizado.^28^ Ele tem vantagens específicas pela sua facilidade de inserção e é uma opção atrativa em hospitais com recursos limitados. Esse dispositivo também facilita o transporte de pacientes para centros com intervenções mais avançadas.^29^

A dislipidemia ocorreu em 46,9% dos pacientes da amostra, sendo observada significância estatística com p = 0,024 (OR: 0,19; IC 95%: 0,05 a 0,81). A identificação desse fator de risco, que foi referido pela população estudada, estava relacionada com uso de estatina e não com avaliação laboratorial da dosagem sérica do colesterol e suas frações. Pacientes com valores de HDL-c < 35 mg/dL têm risco mais elevado. Entretanto, quando os valores são > 60 mg/dL, há um efeito protetor.^30^ Haveria correlação do achado desse dado de efeito com a redução da mortalidade na nossa amostra por estarem em uso de medicação e, portanto, isso configuraria esse efeito de proteção?

### Limitações

Por se tratar de um estudo observacional de longa duração, abrangendo 17 anos de informações, o viés desse trabalho consiste em mudanças nos padrões de prontuários médicos, ocasionando ausências de algumas informações específicas para cálculo de escores e variáveis, bem como novas modificações nos critérios de classificação de choque cardiogênico.^26,27^

## Conclusão

Apesar das variáveis analisadas não se associarem à mortalidade intra-hospitalar, demonstramos que a idade aumentou o risco de óbito. O implante do BIA após 1 dia do diagnóstico atuou como fator de redução de risco. A identificação precoce do estado de choque cardiogênico com implante imediato do BIA apresenta importância significativa na redução da mortalidade.
