# Supplementary material for: Preditores de Mortalidade Intra-Hospitalar de Pacientes com Infarto Agudo do Miocárdio com Choque Cardiogênico em Uso de Balão Intra-Aórtico
Source: Arq Bras Cardiol. 2025 Feb 4;122(2):e20230496. [Article in Portuguese] doi: 10.36660/abc.20230496 (PMC11870121; doi:10.36660/abc.20230496)
Supplement: Translation

# Translation: In-Hospital Mortality Predictors in Patients with Acute Myocardial Infarction and Cardiogenic Shock Using Intra-Aortic Balloon Pump

**DOI:** 10.36660/abc.20230496i

**Authors:** Rossana Dall’Orto Elias, Isabella Pedrosa Assunção, Julliane Vasconcelos Joviano Santos, Maria da Gloria Rodrigues-Machado, José Luiz Barros Pena

**Affiliations:** 1 Brazil Biocor Instituto, Nova Lima, MG – Brazil; 2 Brazil Faculdade de Ciências Médicas de Minas Gerais, Belo Horizonte, MG – Brazil; 3 Brazil Hospital Felicio Rocho - Ecocardiografia, Belo Horizonte, MG – Brazil

**Keywords:** Cardiogenic Shock, Intra-Aortic Balloon Pumping, ST Elevation Myocardial Infarction, Hospital Mortality

## Abstract

**Background:**

Patients with ST-segment elevation myocardial infarction (STEMI) and cardiogenic shock (CS) have a high risk of death. New types of mechanical devices have limited availability in Brazil. The use of intra-aortic balloon pump (IABP), although new guidelines downgraded its recommendation, is the most widely used mechanical support strategy. However, little is known about the clinical predictors of its effectiveness in reducing mortality in this group of patients.

**Objectives:**

To assess the predictors of IABP effectiveness in reducing in-hospital mortality in patients with STEMI and CS.

**Methods:**

This observational, retrospective, descriptive, single-center study involved 98 patients with STEMI and CS treated with IABP, in an intensive care unit. We compared patients who survived (42 men and 13 women) and those did not (30 men and 13 women) using clinical predictors of IABP effectiveness in reducing in-hospital death, considering a statistical significance level of 5% (p < 0.05).

**Results:**

The use of IABP in patients less than 1 day after infarction (odds ratio [OR]: 0.12; 95% confidence interval [CI]: 0.02 to 0.85; p = 0.034) was a factor that increased the risk of in-hospital death. Younger age (OR: 1.09; 95% CI: 1.02 to 1.16; p = 0.010) and dyslipidemia (OR: 0.19; 95% CI: 0.05 to 0.81; p = 0.024) were predictors of reduced in-hospital mortality. For each additional year of age, the risk of death increased 1.07-fold.

**Conclusion:**

In patients with STEMI and CS, the use of IABP reduced in-hospital mortality when it was used for 2 or more days, as well as in younger patients and those with dyslipidemia. Additional studies are needed to confirm these findings.

## Introduction

Acute myocardial infarction (AMI) is the most common cause of cardiogenic shock,^1^ with an incidence between 5% and 15%^2^ and an elevated mortality rate of over 50%.^3^

Intra-aortic balloon pump (IABP) remains a widely used support device in several cardiology services, although it is being replaced with increasing frequency.^4,5^ This device assists the heart by indirectly reducing afterload and increasing diastolic pressure at the aortic root. These effects increase coronary blood flow, resulting in better perfusion. The cardiovascular effects of IABP are due to actions on pre- and afterload, decreasing systolic blood pressure by up to 10% and end-diastolic aortic pressure by up to 30%. There is also an increase in left ventricular ejection fraction (LVEF), with an increase in cardiac output between 0.5 and 1 L/min or up to 30%.^6-8^ The mechanism of action of IABP derives from the concept of counterpulsation: diastolic inflation and rapid systolic deflation. The volume increase in the aorta during diastole results in improved coronary circulation with redistribution of blood flow, increasing coronary perfusion. Rapid deflation leads to a reduction in afterload (Figure 1). These mechanisms theoretically provide an increase in oxygen supply while reducing myocardial oxygen consumption.^9,10^

**Figure 1:** – Schematic representation of the IABP in systole and diastole with ECG tracing and corresponding IABP tracings. The device functions to inflate during diastole (right) and deflate during systole. This can be accomplished by timing the ECG or pressure waves, illustrated above, in order to accurately inflate during the appropriate portion of the cardiac cycle. The IABP waveform, illustrated in blue, is timed to correlate vertically with diastole on the arterial and ECG tracings. ECG, electrocardiogram; IABP: intra-aortic balloon pump.

Post-AMI cardiogenic shock has been the main indication for IABP for years. Nonetheless, the results of the 2012 IABP-SHOCK II trial, the largest study related to IABP, led to a significant decline in its use.^5,11^ The study showed that there was no difference between the two groups in relation to all-cause mortality 30 days after AMI or in relation to rates of reinfarction, repeat revascularization, stroke, sepsis, peripheral ischemic complications, renal failure, or major bleeding. Despite the neutral effects of IABP in cardiogenic shock in patients with acute ST-segment elevation myocardial infarction (STEMI), subgroup analysis of the IABP-SHOCK II trial revealed that young patients, patients without previous AMI, and those without hypertension benefited from IABP.

Notwithstanding the emergence of other mechanical circulatory support devices, such as venoarterial extracorporeal membrane oxygenation (VA-ECMO), IABP has technical advantages such as ease of implantation, greater familiarity of the medical team, lower costs, and fewer complications compared to other models.^10^

Even with changes in the guidelines regarding the use of IABP, studies are still needed, given that this device theoretically provides an increase in the oxygen supply/demand ratio, resulting in greater endocardial viability.^6^ The objective of this study was to assess predictors of in-hospital mortality in patients who used IABP in patients with STEMI and to identify subgroups who would benefit from its use.

## Materials and methods

This was an observational, cross-sectional, retrospective, descriptive, and analytical study, conducted at a single center. A total of 98 patients admitted to Biocor Instituto with diagnosis of STEMI between January 2005 and April 2022 were assessed. Patients who developed cardiogenic shock after STEMI and used IABP were included. The exclusion criteria were aneurysmal dilation of the aorta; postoperative surgery of the ascending and descending aorta; presence of moderate to severe aortic insufficiency; patients with post-cardiac arrest who achieved return of spontaneous circulation, but had an unfavorable neurological outcome; isolated right ventricular infarction; severe peripheral arterial disease; and patients with femoral artery bypass graft.

AMI was defined as persistent chest pain with detection of an increase or decrease in the levels of myocardial injury markers (with at least 1 value above the 99th percentile). One of the following 5 criteria had to be present for the diagnosis of infarction to be confirmed: (1) symptoms of myocardial ischemia; (2) new ST-segment/T-wave changes or complete left bundle branch block; (3) development of pathological Q waves on electrocardiogram; (4) loss of viable myocardial muscle or abnormal wall motion on imaging; (5) identification of intracoronary thrombus on angiography or autopsy.^12,13^

Cardiogenic shock was defined clinically as hypotension (systolic blood pressure < 90 mmHg for > 30 minutes or need for continuous administration of vasopressors for > 30 minutes to maintain systolic blood pressure > 90 mmHg despite adequate volume loading, in addition to target organ hypoperfusion [cool extremities or urine output < 30 mL/h]), radiological signs of pulmonary congestion, and elevated serum lactate concentration.^14,15^

The composite characteristics of sex, age, medical history of comorbidities such as systemic arterial hypertension (SAH), diabetes mellitus (DM), smoking, dyslipidemia, previous AMI, family history of coronary disease,^16^ risk scores such as Killip-Kimball classification^17^ and TIMI Risk,^18^ assessment of left ventricular function by echocardiography, assessment of the culprit coronary artery in AMI, door-to-balloon time, duration of use of circulatory support (IABP), and death were included in the collection of medical records.

SAH was defined as systolic pressure > 140 mmHg or diastolic pressure > 90 mmHg during physical examination, or use of antihypertensive medications. DM was defined as fasting glucose > 126 mg/dL, or use of insulin or oral hypoglycemic agents. Smokers were defined as active smokers at the time of hospital admission or those who had stopped smoking within the past 6 months. Dyslipidemia was defined as total serum cholesterol > 200 mg/dL or the use of statins. History of coronary atherosclerotic disease was defined as AMI prior to admission or any previous vascular intervention.^19^

Left ventricular function was assessed by calculating LVEF according to the Simpson method, and the results from the first transthoracic echocardiogram performed on patients with STEMI after hospital admission were collected. Left ventricular dysfunction was defined as LVEF less than or equal to 40%.

Data collection was performed after the project received approval from the Medical Ethics Committee and Research Ethics Committee of the Faculty of Medical Sciences, within the ethical precepts, in full compliance with the research rules for conducting studies, with respect to professional confidentiality and non-disclosure of patients’ identity, causing them no physical or moral harm (CAAE: 49871221.4.0000.5134).

### Statistical analysis

Data were displayed in tables containing the absolute frequencies and their respective percentages, as well as mean ± standard deviation or median and interquartile range for continuous variables with and without normal distribution, respectively. Continuous variables were tested for normality using the Kolmogorov-Smirnov test. For bivariate analysis, considering death as the outcome, the unpaired Student’s t test and Mann-Whitney test were used for the continuous variables of age and door-to-balloon time, respectively. For categorical variables, the chi-square test and Fisher’s exact test were used. Monte Carlo simulation was used for more than 2 response categories at frequencies lower than 5. For all tests, a significance level of 5% was adopted; therefore, comparisons whose p values were lower than 5% were considered significant.

To determine the factors that were jointly associated with death, a backward stepwise multivariate logistic regression model was created. During this stage, all variables that presented a p value < 0.20 in the bivariate analysis were selected for inclusion in the initial multivariate logistic model. The variables that presented a level of statistical significance (p < 0.05) and significant odds ratio according to a 95% confidence interval remained in the final multivariate logistic model. Variables that had more than 2 categories were transformed into “dummy” variables, and variables that showed collinearity were evaluated and removed from the model. The likelihood ratio test was used to define the final model. The model’s performance was assessed using the Hosmer-Lemeshow test.

The analyses in this study were performed using SPSS, version 25.0, in conjunction with Microsoft Excel (spreadsheet editor).

## Results

Between January 2005 and April 2022, we selected 98 medical records of patients who developed cardiogenic shock after STEMI and received IABP, at a single institution in Brazil.

Table 1 displays the characteristics of the study sample. The majority were male (73.5%), and the mean age was 66.5 ± 12.3 years (ranging from 37 to 93 years). The mean door-to-balloon time was 60 ± 25.6 minutes, ranging from 20 to 180 minutes. The most frequent comorbidity among patients was SAH, which was present in 70 patients (73.7%). Smoking and family history corresponded to 37.8% and 31.2%, respectively.

**Table 1:** – Characteristics of study patients

Variables	n (%)
**Sex**	
Male	72/98 (73.5%)
Female	26/98 (26.5%)
**Age (in years)**	
Mean (SD)	66.5 (12.3)
Median	65.5
**Balloon-to-door time (in minutes)**	
Mean (SD)	60 (25.6)
Median	60
**Comorbidities**	
SAH	70/95 (73.7%)
Dyslipidemia	38/81 (46.9%)
Diabetes	34/95 (35.8%)
Prior AMI	12/95 (12.6%)
Smoking	34/95 (37.8%)
**Family history of coronary insufficiency**	24/77 (31.2%)

*Data are shown as mean (SD) and median. AMI: acute myocardial infarction; SAH: systemic arterial hypertension; SD: standard deviation.*

The majority of patients with STEMI who were hypotensive upon admission and underwent IABP implantation were in Killip class IV (39.2%). The most common culprit artery was the anterior descending artery in 80% of patients, and 95 patients (95.9%) developed ventricular dysfunction. The IABP was implanted on the same day of the AMI in 73 patients (74.5%), and most used the device for 3 or more days (46.9%), as shown in Table 1.

Urgent coronary artery bypass graft (CABG) as a form of revascularization was used in 4% of patients (Table 1), demonstrating success in all cases (Table 2). The total percentage of deaths reached 43.9%, and hospital discharge reached 55.7% (Table 1).

**Table 2:** – Characteristics and progression of patients

Variables	n (%)
**Killip**	
I	18/97 (18.6%)
II	27/97 (27.8%)
III	14/97 (14.4%)
IV	38/97 (39.2%)
**Urgent CABG**	4/98 (4.1%)
**Ventricular dysfunction**	57/87 (66%)
**Hospital discharge**	54/97 (55.7%)
**Culprit artery**	
Anterior descending	72/90 (80%)
Circumflex	7/90 (7.8%)
Right coronary	11/90 (12.2%)
**Days from AMI to IABP implantation**	
0 days	73/98 (74.5%)
1 day	14/98 (14.3%)
2 days	4/98 (4.1%)
3 or more days	7/98 (7.1%)
**Duration of IABP use**	
0 to 1 day	13/98 (13.3%)
2 to 3 days	39/98 (39.8%)
3 or more days	46/98(46.9%)
**Death**	43/98 (43.9%)

*Data are shown as absolute numbers and (percentage). AMI: acute myocardial infarction; CABG: coronary artery bypass graft surgery; IABP: intra-aortic balloon pump.*

Figure 2 displays the TIMI risk score classification of the patients assessed, demonstrating that 50% had a result greater than 8 (median = 8; interquartile range = 5 to 10), representing a risk of death within 30 days greater than 26.8% according to the score indices.

**Figure 2:** – TIMI risk score classification.

When analyzing the primary outcome consisting of in-hospital death and correlating it with the variables studied, no statistically significant difference was found (p ≥ 0.05) for sex, door-to-balloon time, prior AMI, DM, SAH, smoking, dyslipidemia, family history of coronary insufficiency, urgent CABG, ventricular dysfunction, culprit artery, and days from AMI to IABP implantation. In other words, these analyzed variables were not associated with the outcome of death (Table 3).

**Table 3:** – Assessment of study variables according to outcome (death)

Variables	Death		p value
	No (n=55)	Yes (n=43)	
**Sex**			
Male	42 (76.4%)	30 (69.8%)	0.496^q^
Female	13 (23.6%)	13 (30.2%)	
Age (years)*			
< 50 years	7	0	0.017^q^
50 to 75 years	35	29	
> 75 years	3	14	
Door-to-balloon time (min)**	50 (45 - 70)	60 (42 - 80)	0.438
**Prior AMI**			
No	45 (84.9%)	38 (90.5%)	0.540^q^
Yes	8 (15.1%)	4 (9.5%)	
**Diabetes**			
No	34 (64.2%)	27 (64.3%)	> 0.999^q^
Yes	19 (35.8%)	15 (35.7%)	
**Systemic arterial hypertension**			
No	12 (22.6%)	13 (31%)	0.482^q^
Yes	41 (77.4%)	29 (69%)	
**Smoking**			
No	31 (62%)	25 (62.5%)	> 0.999^q^
Yes	19 (38%)	15 (37.5%)	
**Dyslipidemia**			
No	19 (44.2%)	24 (63.2%)	0.119^q^
Yes	24 (55.8%)	14 (36.8%)	
**Family history of coronary insufficiency**			
No	24 (60%)	29 (78.4%)	0.092^q^
Yes	16 (40%)	8 (21.6%)	
**Urgent CABG**			
No	51 (92.7%)	43 (100%)	0.129^f^
Yes	4 (7.3%)	0 (90.0%)	
**Killip**			
I, II, and III	38 (70.4%)	21 (48.8%)	**0.031^**q**^**
IV	16 (29.6%)	22 (51.2%)	
**Ventricular dysfunction**			
No	25 (35%)	5 (42%)	0.115^f^
Yes	47 (65%)	7 (58%)	
**Culprit artery**			
Anterior descending	43 (86%)	29 (72.5%)	0.238^mc^
Circumflex	2 (4%)	5 (12.5%)	
Right coronary	5 (10%)	6 (15%)	
**Days from infarction to IABP implantation**			
0 days	37 (67.3%)	36 (83.7%)	0.053^mc^
1 day	12 (21.8%)	2 (4.7%)	
2 or more days	6 (10.9%)	5 (11.6%)	
**Duration of IABP use**			
0 to 1 day	1 (1.8%)	12 (27.9%)	**0.001^**mc**^**
2 to 3 days	25 (45.5%)	14 (32.6%)	
More than 3 days	29 (52.7%)	17 (39.5%)	

AMI: acute myocardial infarction; CABG: coronary artery bypass graft surgery; f: Fisher’s exact test; IABP: intra-aortic balloon pump; mc: chi-square with Monte Carlo simulation; q: Pearson’s chi-square test. * Data shown as frequency; ** data shown as median (P25 to P75).

On the other hand, in relation to duration of IABP use, the death and non-death groups showed a statistically significant difference (p < 0.001); a lower percentage of patients with the outcome of death had 2 or more days of use in relation to patients who used IABP for a period of 0 to 1 day (Table 3).

Table 4 displays the multivariate logistic regression model for the primary outcome consisting of in-hospital death, indicating which factors were jointly associated with the outcome. The initial model presented all variables with a p value < 0.20, except age, dyslipidemia, and days from AMI to IABP implantation.

**Table 4:** – Multivariate regression model for the outcome of death

		Full model			
	B coefficient	p value	OR	95% CI for OR	
Variables					
Age	0.08	0.010	1.09	1.02	1.16
Dyslipidemia	−1.65	0.024	0.19	0.05	0.81
Family history of coronary insufficiency	−0.47	0.529	0.63	0.15	2.69
Urgent CABG	−21.23	0.999	0.00	0.00	
Killip	0.339	0.510	1.40	0.50	3.93
Days after infarction (0 days, reference category)		0.052			
Days after infarction (1 day)	−2.16	0.034	0.12	0.02	0.85
Days after infarction (2 or more days)	0.85	0.456	2.34	0.25	21.87
Duration of IABP use (0 or 1 day, reference category)		0.921			
Duration of IABP use (2 to 3 days)	−20.91	0.999	0.00	0.00	
Duration of IABP use (more than 3 days)	−21.18	0.999	0.00	0.00	
Constant	17.11	0.999	2699.61		
		**Full model**			
	**B coefficient**	**p value**	**OR**	**95% CI for OR**	
Age	0.07	0.005	1.07	1.02	1.13
Dyslipidemia	−1.58	0.005	0.21	0.07	0.63
Days after infarction (0, reference category)		0.008			
Days after infarction (1 day)	−2.91	0.002	0.05	0.01	0.34
Days after infarction (2 or more days)	−0.44	0.579	0.64	0.13	3.07
Constant	−3.69	0.020	0.03		

*Hosmer-Lemeshow test: p = 0.976; pseudo-R = 0.317; percentage of correct classification = 72.8%. CABG: coronary artery bypass graft surgery; CI: confidence interval; IABP: intra-aortic balloon pump; OR odds ratio.*

For each additional year of age, there was a 1.07-fold increase in the chance of death. However, patients with dyslipidemia and patients with IABP implantation 1 day after infarction showed a reduced risk of death, compared to patients who used IABP at the time of diagnosis (day 0), as shown in Table 4.

## Discussion

The main objective of this study was to identify the clinical characteristics associated with the prognosis of IABP use in patients with STEMI who developed cardiogenic shock. Unlike the IABP-SHOCK II study,^1^ which assessed mortality at 30 days and during a 6.2-year follow-up, this study was limited to the in-hospital period, without considering the impact on survival after hospital discharge.

As demonstrated, predictors such as sex, door-to-balloon time, prior AMI, days from AMI to IABP implantation, DM, SAH, smoking, dyslipidemia, family history of coronary insufficiency, urgent CABG, ventricular dysfunction, and culprit coronary artery were not determinants in the impact on in-hospital mortality.

Similar to the CULPRIT-SHOCK trial,^20^ in the present study the anterior descending artery was the most prevalent in cases of cardiogenic shock, probably because it is associated with a large amount of compromised myocardial muscle when occluded. Although this muscle at risk, classified by LVEF, which we denominated ventricular dysfunction in the study, was identified in almost all patients in the cardiogenic shock group, this variable did not represent an impact factor for death.

The TIMI^17^ and Killip-Kimball^17,18^ risk scores were calculated at admission, and IABP implantation was performed after progression to cardiogenic shock. Our data showed that, among patients admitted with cardiogenic shock during STEMI and IABP implantation, 51.4% died, while, comparatively, in the Killip-Kimball score, mortality was 81% with IABP implantation. Similar to the IABP-SHOCK II study,^1^ this study did not specify the severity of the clinical condition or classify the cardiogenic shock of patients who received IABP implantation, which may be related to the high mortality rate at the time of device implantation at diagnosis and the high mortality rate of patients who received IABP for less than 2 days, as demonstrated by the statistical analysis in Tables 3 and 4.

Regarding the time of occurrence of STEMI and IABP implantation, we observed that, in 73 patients (74.5% of the sample), the implantation occurred within the first 24 hours, and 36 of these patients died. These data confirm the findings in the literature regarding high mortality.^21^

In comparison with randomized controlled clinical trials and analyses with counterpulsation devices, as cited in the studies by Vallabhajosyula et al.^11^ and Koenig et al.,^7^ the studies did not show superiority of other devices in relation to IABP, which may be the option of choice, especially in developing countries.

In our sample, we found 43 patients who used IABP for more than 3 days. Mortality was higher in this group than in those who used it for 3 days or less. This result is probably related to the severity of progression and maintenance of cardiogenic shock, with persistent ventricular dysfunction requiring the use of vasopressors.^22^

IABP was introduced into clinical practice 5 decades ago, and it continues to be one of the most commonly used support devices in cardiogenic shock in Brazil.^23^ IABP is believed to decrease myocardial oxygen consumption, increase coronary artery perfusion, decrease afterload, and modestly increase cardiac output (0.8 to 1 L/min).^22^ There are several ventricular assist devices; however, the most commonly used in cardiogenic shock are the Impella and IABP devices. Impella acts independently of cardiac function and rhythm, and, as cardiac flow rate increases, it progressively relieves the left ventricle and, consequently, myocardial oxygen consumption.^22^

The IMPRESS in Severe Shock trial randomly compared the use of Impella versus IABP in patients with AMI associated with cardiogenic shock. The primary outcome was 30-day mortality, and the study found no significant difference in 30-day mortality (approximately 50% for both groups). ^24^

The SHOCK,^24^ IABP-SHOCK II,^1^ and IMPRESS studies in severe cardiogenic shock^24^ showed approximately 50% mortality at 6 to 12 months, elucidating the consistent mortality results in cardiogenic shock over the past 2 decades, despite the widespread use of mechanical circulatory support devices. A recent analysis of the cVAD (catheter-based ventricular assist device) registry indicated that early implantation of mechanical circulatory support in patients with cardiogenic shock, before initiating inotropic/vasopressor support and before angioplasty, was independently associated with better survival rates in patients with shock due to AMI.^25^

Data are still lacking in the literature on the clinical and hemodynamic profile of patients who received and would benefit from the use of IABP, in addition to a post-hospital follow-up, aiming not only to assess intra-hospital mortality, but also peri- and post-hospital mortality of cardiogenic shock.

There are still important distinctions to be analyzed in the future to assess the effectiveness of circulatory support devices, such as the severity of cardiogenic shock, with a 5-stage classification model suggested by the Society for Cardiovascular Intervention and Angiography in the United States,^26,27^ as a way to stratify risk and define which patients would benefit from the use of counterpulsation devices. Studies to evaluate and monitor the use of IABP have been published in the medical literature more frequently,^5^ some of which diverge from the large-scale IABP-SHOCK II study, which led to the downgrading of the recommendation for the device in the latest guidelines.^13^ However, these new studies are still lacking in assessment of early device implantation and a clinical and universal definition of the classification of cardiogenic shock to assess factors for improving prognosis and reducing in-hospital and long-term mortality. Although other mechanical circulatory support devices have been developed, IABP continues to be widely used.^28^ It has specific advantages due to its ease of insertion and is an attractive option in hospitals with limited resources. This device also facilitates transporting patients to centers with more advanced interventions.^29^

Dyslipidemia occurred in 46.9% of the patients in the sample, with statistical significance observed at p = 0.024 (odds ratio: 0.19; 95% confidence interval: 0.05 to 0.81). The identification of this risk factor, which was reported by the study population, was related to the use of statins and not to laboratory evaluation of serum cholesterol levels and its fractions. Patients with HDL-c values < 35 mg/dL are at a higher risk. However, when values are > 60 mg/dL, there is a protective effect.^30^ Might there be a correlation between the finding of this effect data and the reduced mortality in our sample due to the use of medication, and would this, therefore, represent this protective effect?

### Limitations

As this was a long-term observational study, encompassing 17 years of data, the bias of consists of changes in the standards of medical records, resulting in the absence of some specific data for calculating scores and variables, as well as new modifications in the criteria for classifying cardiogenic shock.^26, 27^

## Conclusion

Although the variables analyzed were not associated with in-hospital mortality, we demonstrated that age increased the risk of death. Implantation of the IABP 1 day after diagnosis acted as a risk reduction factor. Early identification of the state of cardiogenic shock with immediate implantation of IABP is significantly important in reducing mortality.
